# How Moving Backgrounds Influence Interception

**DOI:** 10.1371/journal.pone.0119903

**Published:** 2015-03-13

**Authors:** Eli Brenner, Jeroen B. J. Smeets

**Affiliations:** Faculty of Human Movement Sciences, MOVE Research Institute, VU University, Amsterdam, The Netherlands; University of Reading, UNITED KINGDOM

## Abstract

Reaching movements towards an object are continuously guided by visual information about the target and the arm. Such guidance increases precision and allows one to adjust the movement if the target unexpectedly moves. On-going arm movements are also influenced by motion in the surrounding. Fast responses to motion in the surrounding could help cope with moving obstacles and with the consequences of changes in one’s eye orientation and vantage point. To further evaluate how motion in the surrounding influences interceptive movements we asked subjects to tap a moving target when it reached a second, static target. We varied the direction and location of motion in the surrounding, as well as details of the stimuli that are known to influence eye movements. Subjects were most sensitive to motion in the background when such motion was near the targets. Whether or not the eyes were moving, and the direction of the background motion in relation to the direction in which the eyes were moving, had very little influence on the response to the background motion. We conclude that the responses to background motion are driven by motion near the target rather than by a global analysis of the optic flow and its relation with other information about self-motion.

## Introduction

It is long known that people use visual information to guide their on-going goal-directed movements (e.g. [1,2]). Doing so improves performance, both because the time over which one must predict the target’s or one’s own movements decreases during the movement, so that the consequence of errors in (for instance) judging the target’s speed also decrease, and because the movement may have to be changed to be successful, for instance because the target unexpectedly tried to escape [3]. Many studies have demonstrated that visual information is used to guide on-going movements by showing that people respond to externally imposed changes. People respond to changes in target position [4,5], motion [6] and orientation [7–10], as well as to changes in feedback about the position of their hand [11,12] or of a tool that is manipulated by moving the hand [13]. In accordance with the idea that visual information normally continuously guides the hand, people respond to changes even if they do not notice the changes [5,14–16]. Moreover, they are unable to suppress such responses [17–19], probably because the latency with which movements are adjusted is shorter than the time it takes to evaluate whether the response is adequate.

Goal-directed arm movements are not only influenced by displacing the target or the feedback about the position of the hand, but also by presenting motion in the surrounding. Background motion influences arm movements towards visible targets [4,20,21] as well as towards remembered positions [22–24]. Arm movements are influenced both when the whole background moves in a consistent manner [21,23] and when static structures [25] or different motion [26] is visible elsewhere. They are influenced when the target is static [4] or is assumed to be static when it is not visible [23], and sometimes also when the target is moving [27,28].

Why would motion in the background influence our actions? Perhaps motion in the background is attributed to us having moved, so that our actions are adjusted to compensate for such self-motion [29]. The way we walk is influenced by motion in the background that suggests that we are walking at a different speed [30], and targets appear to move faster if the background’s motion suggests that our eyes are moving faster [31]. Attributing global background motion to self-motion is consistent with generally assuming that the world around us is stable [32]. If background motion is attributed to self-motion, the hand should respond to such motion, especially if the target disappears before the background starts to move so that there is no feedback to indicate that it is ‘wrong’ to do so (although the hand also temporarily follows background motion if the target remains visible [4]).

The response to background motion is not just the result of tracking recognizable items near the position at which the target had been, because it is also found when the background consists of randomly distributed dots that are each only visible for a short period of time [23]. Moreover, it also occurs when the region near the endpoint of the arm movement is occluded by a static grey disk, so that the background motion is quite far from the point that is fixated, and towards which the hand is moving [22]. The deviation of the arm in the direction of background motion increases with target speed [33], up to a limit that depends on the spatial frequency [34]. Thus, the response appears to rely on a global processing of motion in the scene.

When intercepting moving targets, a background moving parallel to the target’s direction of motion had little effect [35], and a background moving orthogonal to the target’s motion gave rise to a deviation of the hand in the opposite direction than the background’s motion [27,28], in accordance with the background’s influence on the target’s perceived direction of motion [36]. Why should the hand not simply follow the moving background in such interception tasks? Perhaps pursuing the moving target with one’s eyes changed matters by making it more difficult to separate the motion on the retina into components caused by eye movements, by changes in the vantage point and by object motion, thereby making it more difficult to determine an appropriate response. To make the required distinctions one would have to decompose the optic flow [37,38] or consider vestibular information [39] and information from the eye muscles [31].

Even when moving towards static targets, some responses to non-target motion are less easily interpreted in terms of self-motion. For instance, the hand quickly follows local motion of an obstacle in an otherwise static scene, even if doing so is counterproductive [25]. The tendency to respond to the obstacle’s motion might have been enhanced by the obstacle not being irrelevant to the task. However, if two large task-irrelevant structures move in opposite directions, the hand follows the one closest to the target [26]. Thus, the hand might simply follow any motion in the vicinity of the target. We therefore decided to further examine how the influence of background motion depends on eye movements (Experiments 1 and 2) and on which parts of the background move (Experiment 3).

## Methods

**Fig 1:**
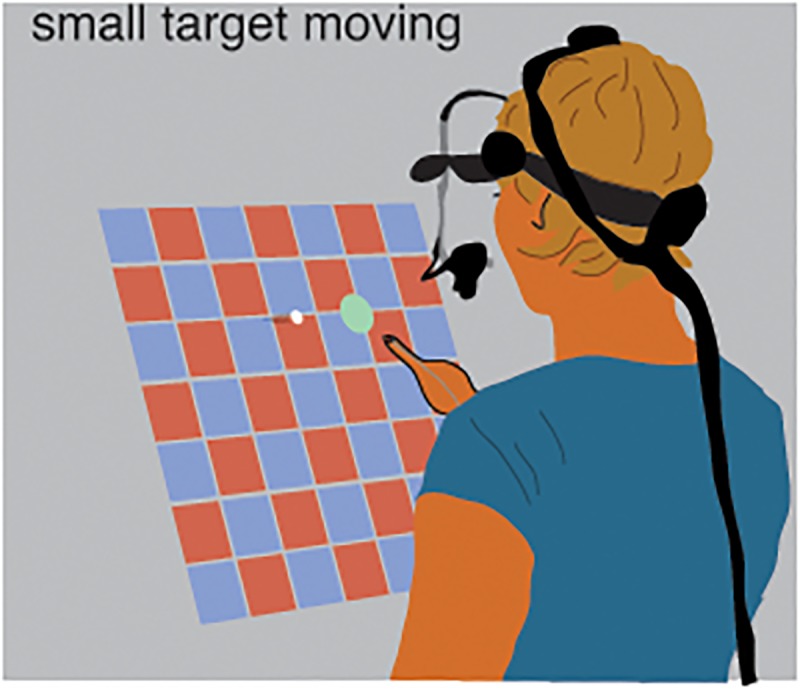
Schematic representation of the task. The subject was standing in front of a slanted screen and tried to tap on a moving, white target when it was aligned (superimposed) with a static, green target. Eye movements were recorded with a head-mounted eye tracker.

In previous studies, the arm responded to background motion after about 150 ms, and continued to follow the background for about 100ms, so the hand’s deviation from its usual trajectory was maximal about 250 ms after the background started to move [4,34]. We therefore designed our experiments in such a manner that we would know approximately when our subjects would hit the targets, and moved the background 250 ms before this moment to get a maximal displacement at the moment of the hit. The task was to tap on virtual targets with one’s right index finger. On each trial there was one target that moved rightward across a screen and another target that was static on the moving target’s path (Fig. 1). Subjects were instructed to hit the moving target at the moment that it was aligned with the static target, or equivalently, to hit the static target at the moment that the moving target was aligned with it. Thus both the time and place at which one was expected to tap were fixed. We influenced our subjects’ eye movements indirectly, by adjusting the sizes of the two above-mentioned targets. One was always much smaller than the other. We expected the eyes to be directed at the smaller target [3], and therefore to pursue the moving target when it was small, but not when it was large (and the static target was small).

### The subjects

Ten subjects, including the two authors, took part in Experiment 1 (4 male, 6 female, 25–55 years of age). Ten subjects took part in Experiment 2, including one author and one of the other subjects who had taken part in Experiment 1 (5 male, 5 female, 24–56 years of age). Twenty-one subjects took part in Experiment 3, including all subjects who had taken part in Experiment 2, four who had taken part in Experiment 1 but not in Experiment 2 (including the other author), and seven subjects who had not taken part in either of the previous experiments (11 male, 10 female, 24–56 years of age). There were two sessions per subject in Experiments 1 and 2, and a single session per subject in Experiment 3. Only the authors were explicitly aware of the manipulations under study, but most of the manipulations were quite evident. The study is part of a research program that has been approved by the *Ethische Commissie Bewegingswetenschappen* of the VU University. All subjects signed the standard informed consent forms.

### The setup

The experiments were conducted in a normally illuminated room (fluorescent illumination). Images were presented at 120 Hz (InFocus DepthQ Projector; resolution: 1280 by 768 pixels). They were projected from behind onto a 1.00 m by 1.25 m (height by width) acrylic rear-projection screen (Techplex 150) that was tilted backwards by 30°. Subjects stood in front of the screen and tapped the screen with their right index finger. They were not restrained in any way. An infrared camera (Optotrak 3020, Northern Digital) that was placed at about shoulder height to the left of the screen measured the position of a marker (an infrared light emitting diode) attached to the nail of the subject’s right index finger at 500 Hz. In Experiment 1, a head-mounted eye tracking system (Eyelink II, SR Research) measured the movements of both eyes, also at 500 Hz, and the Optotrak also measured the positions of three markers that were attached to the left side of the eye tracking system to estimate the subject’s head movements.

In order to synchronize the movement registration with the stimulus presentation, a light sensor was placed in the path of the light directed towards the top left corner of the screen. A custom-built electronic circuit ensured that either one of the markers attached to the eye tracker (Experiment 1) or a marker that was attached to the left side of the screen (Experiments 2 and 3) was switched off for about 10ms when light fell on the sensor. The delay between light falling on the sensor and the marker being switched off was 1ms. How this was used to synchronize the movement data with the stimulus presentation will be explained in the next section.

### Calibration

At the beginning of each session, the position of the marker on the fingertip was measured when the fingertip was at four indicated positions on the screen. This simple four-point calibration was used to relate the position of the fingertip to the projected images, automatically correcting for the fact that the marker was attached to the nail rather than the tip of the finger. Although new images were presented only every 8.3ms, we determined the moments at which these images were presented with respect to the measurements of the finger (and head) to within 2ms by presenting flashes at the top left corner of the screen whenever a new target appeared. We interpolated these positions to estimate the target position every 2ms, in synchrony with the registration of the finger position.

To calibrate the eye movement recordings (Experiment 1), subjects were instructed to look at the four indicated positions on the screen while they were moving their fingertip to them for the calibration. This allowed us to combine the eye tracker’s output when looking at these positions with the measured position and orientation of the head at that moment. We used this information to calibrate the eye tracker. During the experiment, when light fell on the sensor directed towards the top left corner of the screen, a signal was also sent to the parallel port of the eye tracker with which we could later synchronize the eye and hand measurements.

### Stimulus and procedure

Targets were presented on a checkerboard-patterned background. The checkerboard pattern consisted of 7 rows and 7 columns of alternating red and blue squares, separated by dark rims of 10% of the squares’ widths. The pattern filled a 50 by 50 cm area at the centre of the screen. Subjects started each trial by placing their index finger at the starting point: a 1 cm diameter yellow disk that was 10 cm to the right of and 10 cm below the screen centre (and therefore also 10 cm to the right of and 10 cm below the centre of the checkerboard). Subjects could rest whenever they wanted to, by not placing their finger at the starting point. Between 1.0 and 1.5 s after the finger was placed at the starting point, the starting point disappeared and two target disks appeared 10 cm above the screen centre. One appeared 20 cm to the left of the screen centre. It was white and was moving to the right at either 40 or 50 cm/s. The other appeared 10 cm to the right of the screen centre. It was green and remained static.

During half of each session the moving target was small (1.4 cm diameter disk) and the static target was large (4.2 cm diameter disk). During the other half the moving target was large and the static target was small. Based on our previous studies [3], we expected subjects to direct their gaze towards the smaller target, irrespective of whether or not it moved. This method of manipulating gaze has the advantage that subjects can perform the interception task without considering secondary requirements such as fixating an indicated location, but it means that not only the eye movements but also the images on the screen are slightly different for the two kinds of trials.

Subjects were instructed to keep their finger on the starting point until the targets appeared. If they lifted their finger too soon, the targets did not appear. In that case, they had to place their finger back at the starting point. The targets then only appeared if they kept their finger at the starting point for between 1.0 and 1.5 s from when the finger was placed back at the starting point. Once the targets appeared, subjects were to lift their finger off the screen and tap on the targets at the right moment (i.e. when the targets’ centres coincided). This ‘right’ moment was 600 ms after the targets appeared if the moving target was moving at 50 cm/s. It was 750 ms after the targets appeared if the moving target was moving at 40 cm/s. We defined the finger’s movement onset as the moment at which it had moved 5mm from its position when the target appeared.

A tap was detected on-line if the reduction in the distance to the screen between consecutive measurements decreased by more than 1 mm (i.e. a deceleration threshold of 250 m/s^2^) while the finger was less than 5 mm above the screen and within 2.1 cm of the target’s path. If the position of the fingertip (as determined during calibration) was within the outline of a target at the moment of the tap, we considered that target to have been hit. All the delays in our equipment were considered when determining the target’s position at the moment of the tap. If a target was hit, it disappeared. If both targets were hit, a sound indicated that the tap had been successful. If the static target was missed, it remained visible for another 500 ms. If the moving target was missed, it deflected away from the finger at 1 m/s, also remaining visible for another 500 ms. Thus, for instance, if the finger tapped within the static target but above and to the left of the moving target, the static target disappeared and the moving target moved down and to the right for another 500 ms (after the tap) before disappearing.

Our main experimental variable was background motion. Details about how the background moved in the three different experiments will be given below. Since the task was to tap the targets at predefined times, we could move the background at a fixed time with respect to the expected time of the tap. We moved the background at the time at which its motion was expected to have a maximal effect on the hand’s position at the moment of the tap. On trials in which elements of the background moved, they always did so at 45 cm/s between 250 and 150 ms before the anticipated time of the tap. Subjects could stand and move in front of the screen in any way that they felt would help them perform the task, so all measurements are presented in cm rather than degrees, because the latter differed between subjects and even between trials.

### Experiment 1

In this experiment we primarily wanted to determine whether eye movements influence how the moving arm responds to background motion. Each of the ten subjects performed two sessions of 240 trials each. In one session, there was a block of 120 trials in which the moving target was small, followed by a block of 120 trials in which the moving target was large. In the other session the order of the blocks was reversed. Half the subjects started with one session and the other half started with the other. Within each group of 120 trials, there were 20 randomly interleaved replications of each of six conditions. The different conditions were all combinations of the two target speeds (40 or 50cm/s) and three background motions (up, down or none). If the background moved, it moved as a whole at a constant speed of 45cm/s, thus moving 4.5cm during the 100ms of background motion. As already mentioned, we expected the eyes to pursue the moving target when it was small, and to fixate the static target when it was small. We were mainly interested in whether the influence of vertical background motion on the vertical tapping errors would also depend on which target was small; i.e. on whether the eyes were pursuing the moving target.

### Experiment 2

In this experiment we examined whether the arm responds differently to sudden background motion parallel to the moving target’s motion, rather than to sudden background motion orthogonal to the moving target’s motion, because previous interception studies found qualitative differences between the influences of continuous background motion parallel to and orthogonal to the target’s motion [27]. We also examined whether there was a quantitative asymmetry between the influence of background motion opposite the direction of target motion and in the direction of target motion, as has previously been found for perceived speed [31]. If such an asymmetry is related to pursuit, it will be particularly strong when the moving target is small. This experiment was identical to the first, other than that the background sometimes moved to the left or right, rather than sometimes moving up or down. We did not examine the eye movements.

### Experiment 3

**Fig 2:**
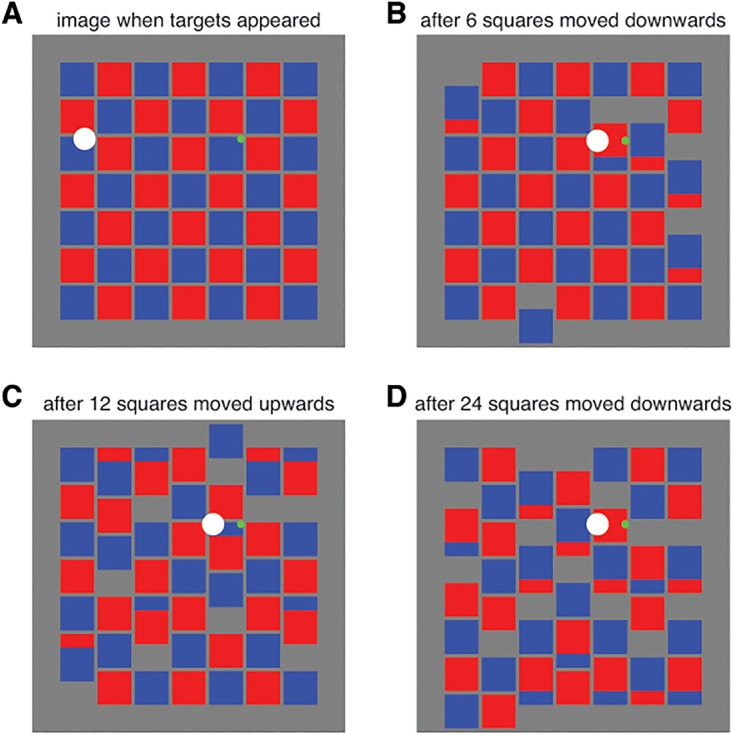
Schematic representation of possible images on the screen in Experiment 3. The images are all for trials in which the moving target was large. The moving target is white, the static target is green, and the background consists of red and blue squares. **A**. The image at the moment that the targets appeared. **B**. An image after 6 squares moved downwards. **C**. An image after 12 squares moved upwards. **D**. An image after 24 squares moved downwards.

In this experiment we wanted to determine whether movements of the arm are particularly susceptible to motion in certain parts of the background. To do so, we moved only a fraction (6, 12 or 24) of the 49 tiles. All the background squares that moved either moved upwards together or downwards together (Fig. 2). Moving squares always occluded stationary ones when they overlapped. Each subject performed a single session with two sets of 120 trials. Ten subjects started with a set in which the moving target was large and eleven subjects started with a set in which the moving target was small. Within each set of 120 trials, there were 10 trials of each of the 12 conditions: two target speeds (40 or 50 cm/s), two background motions (up or down) and three numbers of squares that moved (6, 12 or 24). On each trial the squares that moved were chosen at random.

### Analysis

For the data-analysis, we re-determined the moment of the tap on the basis of the peak acceleration of the fingertip in the direction orthogonal to the screen [40]. By doing so, we improved our measures of tapping errors and managed to include taps that did not reach the acceleration threshold that we used for the on-line detection of taps, so that we could use almost all trials (only occasional trials in which the subject did not move at all or rotated their finger so that the marker was no longer visible at the end of the movement were excluded from further analysis). Acceleration was determined from three consecutive measurements of the distance from the screen (by subtracting the difference between the last two distances from the difference between the first two distances, and assigning the outcome to the moment of the central measurement). This method of determining the moment of the tap was not used during the experiments themselves, because doing so would introduce additional delays in providing the feedback.

To get an indication of the time-course of the effects of the background we compared the mean vertical (Experiment 1) or horizontal (Experiment 2) position of the finger in trials in which the background moved, with the mean position in trials in which the background did not move. This was done for each moment during the last 300 ms before the targets were aligned. It was done separately for each subject and direction of background motion (pooled over moving target size and speed), and the values were then averaged across subjects. For Experiment 1, we examined the time course of the change in gaze in the same way (also pooling over the two eyes). Eye movements were expressed as changes in gaze position on the screen. These changes in gaze were determined by combining the measured eye rotations with respect to the head with movements of the head (both rotations and translations). We defined saccades as times at which gaze moved faster than 200cm/s for at least 20ms.

The main measure of interest in Experiment 1 was the systematic effect that vertical motion of the background had on the vertical position at which the finger tapped the screen. For Experiment 2 it was the systematic effect that horizontal motion of the background had on the horizontal position of the tap. For each of these experiments, the mean position was determined for each subject and condition, and subjected to a repeated measures analysis of variance (with factors background motion, target speed and whether the moving or static target was small). Systematic effects on the timing of the tap (with respect to tapping at the moment that the centres of the two targets were aligned) were subjected to a similar analysis, as was the mean gaze velocity (during the time at which the background could move) in Experiment 1. Repeated measures analyses of variance with factors background motion, target speed and whether the moving or static target was small, were also conducted for the standard deviations of the horizontal positions of the taps on the screen (horizontal position), for the standard deviations of the horizontal positions of the taps with respect to the moving target (interception), and for the standard deviations of the timing of the taps.

In Experiment 3, our main interest was in whether the positions of the squares that moved mattered. Since many squares moved on each trial, we determined the relative influence of each square by comparing trials in which that square did move with trials in which it did not. For each subject, moving target size, number of squares that moved, and direction in which the squares moved, we determined the mean vertical position of the tap. We did so separately for the trials in which the square in question was one of the squares that moved, and for the trials in which it was not. We then determined the difference between the pairs of mean vertical positions of the taps for trials in which squares moved upwards and downwards. We divided these differences by two to estimate the mean influence of moving the background (either upward or downward). Finally, we subtracted the difference between the taps when the square in question did not move from the difference when it did move to obtain an estimate of the relative influence of a single moving square. If the hand followed the background more when that square was one of the squares that moved, than when it was not one of the squares that moved, the relative influence of that square was considered positive.

In this manner, we determined each square’s relative influence for each subject, number of squares that moved, and size of the moving target. We did not do so separately for the two target speeds, because we did not expect target speed to matter (variations in target speed were included in the design to match the other experiments and to make sure that people need visual information about target motion to intercept the target). Some squares never moved up or down for some subjects in some of the 6 combined conditions of Experiment 3. In such cases (about 1.7%) we used the average value of all neighbouring squares (up to 8 values) that moved in the direction in question (for that subject in that combined condition) to calculate the square’s relative influence. The relative influences of the individual squares were analysed with a repeated measures analysis of variance, with the factors *background square* (49 values), *number of squares that moved* (6, 12 or 24) and *whether or not the moving target was small*. We also checked whether the number of squares that moved made a difference to the magnitude of the influence of the background by plotting this magnitude as a function of the number of squares that moved. For all our analyses of variance, we report all main and interaction effects that have a *p*-value that is smaller than 0.05.

## Results

### Experiment 1

On average, the finger left the starting position 322 ms after the target appeared for fast targets and 364 ms after the target appeared for slow targets (F_1,9_ = 8.0; *p* = 0.02). This left 278 ms to reach the fast targets and 386 ms to reach the slow targets. The reaction time did not depend on which target was small.

**Fig 3:**
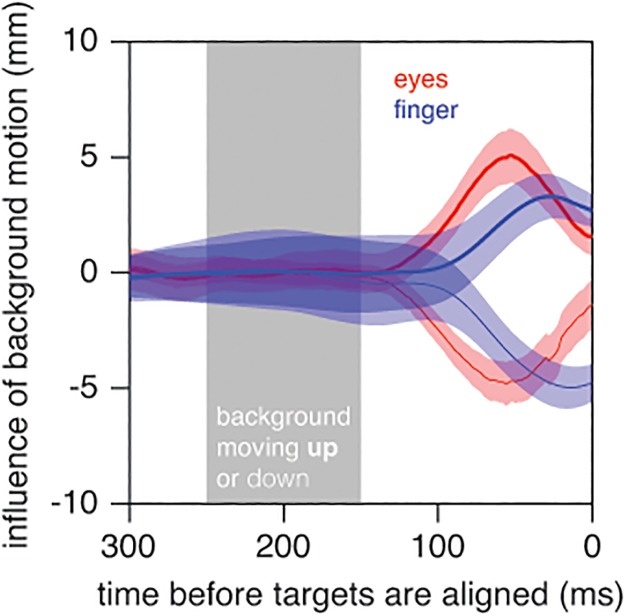
Time course of the influence of background motion in Experiment 1. Response of the finger (blue) and eyes (red) to upward (thick curves) and downward (thin curves) background motion. Positive values are upward (parallel to the screen). Each curve shows the difference between the mean vertical position on trials with and without background motion, averaged across subjects. Shaded regions show the standard error across subjects. The grey area indicates when the background was moving.

Both the finger and the eyes responded to the background’s motion (Fig. 3). The eyes started following the background about 120 ms after it started moving and the hand did so about 150 ms after it did so. The effect on the eyes was similar in magnitude to that on the finger, but occurred a bit earlier. This could be due to the shorter distance from the brain to the eye muscles than from the brain to the muscles of the arm, possibly with a smaller number of synapses, but it could also result from mechanical differences and differences in force generation dynamics [23]. It would appear from the blue curves in Fig. 3 that we would have obtained a slightly larger effect of the background on the tapping errors if the background motion had started about 25 ms later. The eyes’ responses to the moving backgrounds will have influenced the motion of the targets’ and background’s retinal images, but note that any responses to such retinal motion would certainly be too late to influence the tap.

**Fig 4:**
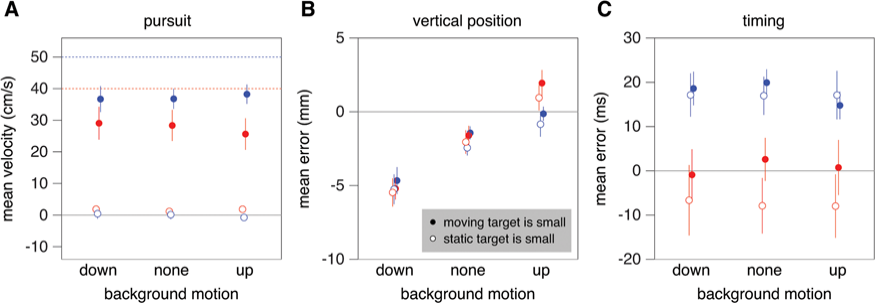
Mean values for Experiment 1. Blue symbols: faster targets; red symbols: slower targets. All symbols show means and standard errors across subjects. **A**. Mean horizontal velocity of the eye during the time period at which the background sometimes moved. As anticipated, the eyes pursued the moving target when it was small (solid symbols), but not when it was large (open symbols). The dashed lines indicate the target speed. **B**. Mean vertical position of the tap (i.e. the error parallel to the background motion). **C**. Timing error (i.e. the position of the moving target with respect to the static target at the time of the tap, divided by the target’s speed).

Switching the sizes of the two targets has a clear influence on the velocity of ocular pursuit (F_1,9_ = 62.4; *p*<0.001; Fig. 4A). The eyes fixated the static target when it was small (and the moving target was large; open symbols). They generally pursued the moving target when it was small (and the static target was large; solid symbols). There was a significant interaction between target speed and which target was small (F_1,9_ = 5.2; *p* = 0.05), because the velocity of pursuit was larger for faster target motion when the moving target was small, but not when it was large (because in the latter case subjects fixated the small static target). When the moving target was small, the mean eye velocity was lower than the moving target’s speed (filled dots below the dashed lines of the same colour), because subjects did not always pursue the moving target. However, it is evident that we managed to influence our subjects’ eye movements by manipulating the two targets’ sizes, as we had anticipated.

To get a general impression of where subjects were looking throughout the trials we also evaluated our subjects’ saccades. We did not instruct our subjects where to look at the beginning of the trial, but the starting point was small enough to always make them look at it when moving their finger to it to start the trial. They then made a saccade upwards to the targets. In 99% of the trials this saccade took place before the targets had appeared. In the blocks of trials in which the moving target was small, 55% of these saccades were to where the moving target would appear and 45% were to where the static target would appear. On average there were only 0.6 additional saccades per trial (including catch-up saccades and saccades to the moving target after an initial saccade to the static target). There were saccades *while the background was moving* in 9% of the trials in which the moving target was small. In the blocks of trials in which the moving target was large, 87% of the first saccades were to where the static target would appear and only 13% were to where the moving target would appear. On average there were only 0.2 additional saccades per trial (one every five trials). There were saccades *while the background was moving* in 1% of the trials in which the moving target was large.

The main question was whether or not the subjects’ gaze behaviour would affect how background motion influenced the position that they tapped (Fig. 4B). We found a significant influence of background motion on the mean vertical error (F_2,18_ = 37.3; *p*<0.001), confirming that the background influenced the arm movements. We also found a significant interaction between background motion and target speed (F_2,18_ = 9.1; *p* = 0.002): the background’s influence was slightly smaller for the faster targets. The influence of background motion did not depend on which target was small (i.e. on the eye movements; F_2,18_ = 0.2; *p* = 0.8). There was a tendency to hit slightly below the target when the background did not move. This could suggest that subjects wanted to be sure not to overshoot the target when moving upwards from the starting position, but it is also possible that they systematically held their finger differently when tapping on the targets than during calibration, either because the force at impact was higher when tapping or because it was beneficial to remain a bit lower when tapping in order to be sure to see the target.

We found a systematic difference between the mean timing errors for the slower and faster targets (F_1,9_ = 12.8; *p* = 0.006; Fig. 4C). Subjects tended to hit too late (positive errors) for the faster targets. This is consistent with the results of previous interception studies that have shown that people consider the target’s properties [41] and their own performance [40] on previous trials to supplement perceptual information from the current trial.

**Fig 5:**
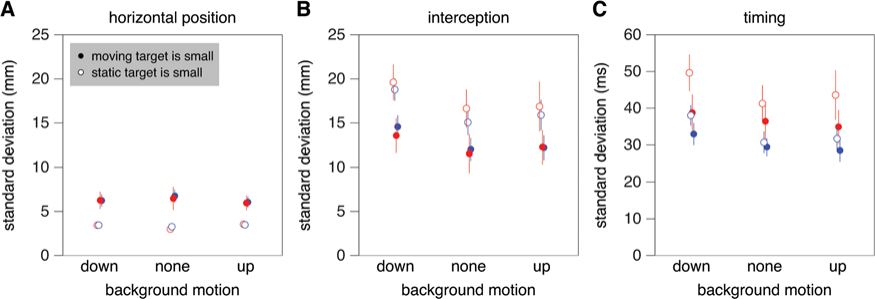
Measures of variable error for Experiment 1. **A**. Horizontal variability of the position of the tap with respect to the static target. **B**. Horizontal variability of the position of the tap with respect to the moving target. **C**. Variability in the time of the tap with respect to when the two targets were aligned. Details as in Fig. 4.

We expected directing one’s gaze at one of the two targets to reduce the variable error with respect to that target, and following the moving target with one’s eyes to reduce the variability in the timing [3]. The standard deviation in where subjects tapped relative to the static target was indeed significantly smaller when the eyes fixated the static target (static target small; F_1,9_ = 13.7; *p* = 0.005; Fig. 5A).

In accordance with our expectations, the standard deviation in where subjects tapped relative to the moving target was significantly smaller when the eyes pursued the moving target (moving target is small; F_1,9_ = 10.8; *p* = 0.01; Fig. 5B). It was also influenced by the background’s motion (F_2,18_ = 13.9; *p*<0.001): performance was more variable when the background moved, especially when it moved downward.

The variability in timing was generally smaller when the eyes pursued the moving target (moving target is small), as expected, but this effect was not statistically significant (Fig. 5C). There was, however, a significant interaction between background motion and which of the two targets was small (F_2,18_ = 4.3; *p* = 0.03); the variability in timing was more evidently smaller when the eyes pursued the moving target if the background moved. There was also a main effect of background motion (F_2,18_ = 14.6; *p*<0.001); again, performance was most variable when the background moved downward. The standard deviation in the timing was also significantly smaller for the faster than for the slower targets (F_1,9_ = 9.8; *p* = 0.01). This suggests that at least part of the variability in the timing has a spatial origin, because the same spatial variability in the horizontal position of the moving target with respect to the static target corresponds with a smaller temporal variability for faster target motion.

### Experiment 2

**Fig 6:**
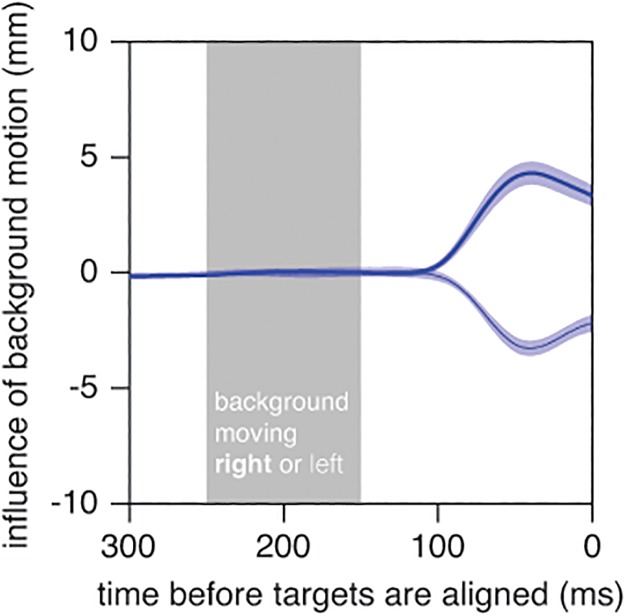
Time course of the influence of background motion in Experiment 2. Response of the finger to rightward (thick curve) and leftward (thin curve) background motion. Positive values are to the right. Further details as in Fig. 3.

On average, the finger left the starting position 311 ms after the target appeared for fast targets and 343 ms after the target appeared for slow targets (F_1,9_ = 10.0; *p* = 0.01). This left 289 ms to reach the fast targets and 407 ms to reach the slow targets. Again, the reaction time did not depend on which target was small. During its movement, the finger responded to the background’s motion in much the same way as it had in Experiment 1 (Fig. 6), so qualitatively the response of the hand to background motion was not different for motion parallel to the target’s motion than for motion orthogonal to the target’s motion. The variability between subjects was much smaller in this experiment than in Experiment 1, probably because the background moved horizontally rather than vertically in this experiment, so that the influence of background motion was also measured in the horizontal rather than the vertical direction. Since the finger only had to move vertically to reach the static target, measurements in the vertical direction (Experiment 1) were influenced by differences in the way subjects perform the main, vertical movement, whereas measurements in the horizontal direction (Experiment 2) were not.

**Fig 7:**
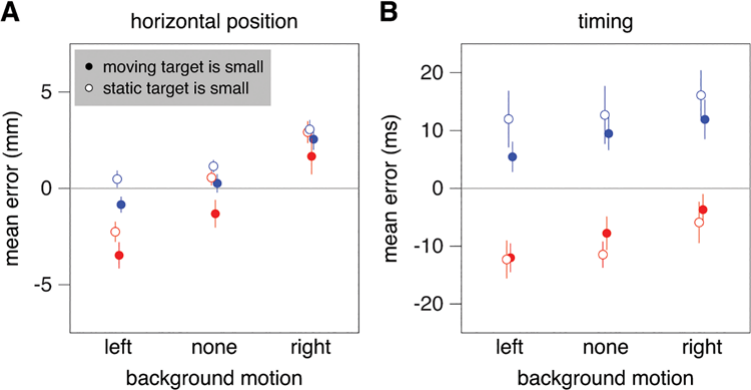
Mean values for Experiment 2. **A**. Horizontal position of the tap with respect to the static target (i.e. the error parallel to background motion). **B**. Timing error (i.e. the position of the moving target with respect to the static target at the time of the tap, divided by target speed). Details as in Fig. 4.

Moving the background to the left did not influence the hand more than moving it to the right (Figs. 6 and 7A). The background’s motion did influence the mean horizontal position of the tap (F_2,18_ = 46.1; *p*<0.001), as did the target’s speed (F_1,9_ = 19.3; *p* = 0.002). Subjects tapped further to the right when the background moved to the right and when the target moved faster. The influence of background motion did not depend on which target was small (interaction effect: F_2,18_ = 0.4; *p* = 0.7). We found a significant interaction between background motion and target speed (F_2,18_ = 16.7; *p*<0.001). Again, the background’s influence was slightly smaller for the faster targets.

Again, we found that subjects tended to hit too late for the faster targets (F_1,9_ = 41.0; *p*<0.001; Fig. 7B). They also tended to hit later when the background moved to the right and earlier when it moved to the left (F_2,18_ = 17.9; *p*<0.001). This could be because in order to intercept the moving target further to the left you have to hit sooner, and in order to intercept the moving target further to the right you have to hit later. However, the effect could also be mediated by an influence of background motion on the perceived target speed. There was a significant interaction between target speed and which target was small (F_1,9_ = 6.0; *p* = 0.04): the differences between the errors for the two target speeds were smaller when the moving target was small (solid red and blue symbols closer together than open red and blue symbols). This is consistent with pursuing the target providing additional information about the target’s speed, so that less weight is given to experience from previous trials.

**Fig 8:**
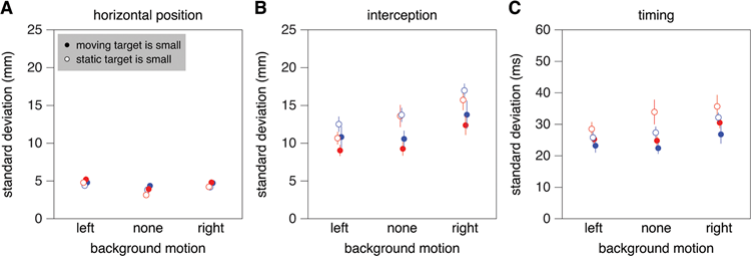
Measures of variable error in Experiment 2. **A**. Horizontal variability of the position of the tap with respect to the static target. **B**. Horizontal variability of the position of the tap with respect to the moving target. **C**. Variability in the time of the tap with respect to when the two targets were aligned. Details as in Fig. 4.

In this experiment, there was a significant influence of background motion on the standard deviation in where subjects tapped the screen (F_2,18_ = 14.8; *p*<0.001; Fig. 8A). The variability was smallest when the background did not move. Pursuing the moving target with the eyes did not reduce precision significantly.

There was a significant influence of both background motion (F_2,18_ = 35.8; *p*<0.001) and target speed (F_1,9_ = 9.0; *p* = 0.02) on the standard deviation in where subjects tapped relative to the moving target (Fig. 8B). The variability was larger when the background moved to the right and smaller for slower targets. There was also a significant interaction between background motion and which target was small (F_2,18_ = 4.5; p = 0.03); the expected improvement in precision when the eyes pursued the moving target was largest when the background did not move.

There was a significant influence of both background motion (F_2,18_ = 23.1; *p*<0.001) and target speed (F_1,9_ = 9.6; *p* = 0.01) on the standard deviation in timing (Fig. 8C). The variability was larger when the background moved to the right and smaller for faster targets. There was also a significant interaction between background motion and which target was small (F_2,18_ = 4.3; p = 0.03); again, the improvement in precision when the eyes pursued the moving target was largest when the background did not move.

### Experiment 3

**Fig 9:**
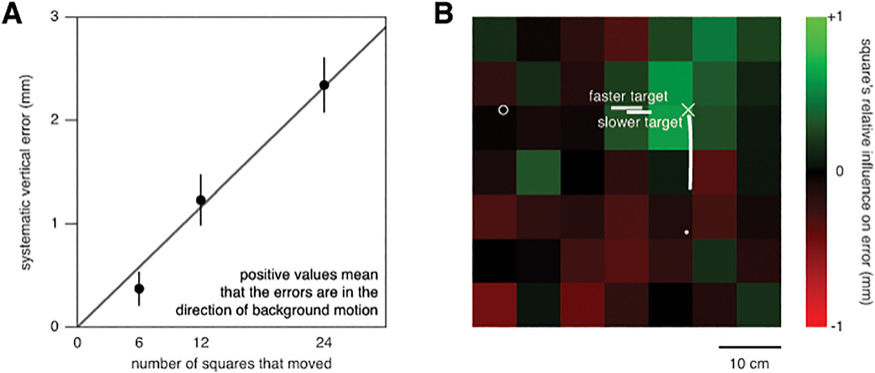
Spatial properties of the effect of vertical background motion in Experiment 3. **A**. How the number of squares that moved influenced the extent to which the vertical position of the tap depended on the direction of background motion. The systematic error is half the difference between the average vertical positions of the taps for upward and downward background motion (tapping above the target centre is considered a positive error). Symbols show the means and standard errors across subjects. The line is a linear fit to the three points (constrained to pass through the origin, because when none of the squares move there can be no difference between upward and downward motion, so if the relationship is linear it must pass through the origin). **B**. The relative influence of each square: the extent to which the tap was further (green) or less far (red) in the direction of background motion when that particular square was one of the squares that moved, than when it was not. The white cross indicates the position of the centre of the static target. The white circle on the left indicates where the moving targets appeared. The horizontal bars indicate the lateral positions of the centres of the moving targets during the time that the background was moving. The white disk is the finger’s starting position. The slightly curved vertical trace shows the projection on the screen of the average displacement of the fingertip while the background was moving.

In this experiment we examined the spatial properties of the influence of background motion. On each trial, we moved 6, 12 or 24 randomly selected squares. Moving only part of the squares, while the other squares remained visible, influenced the taps (Fig. 9A). The magnitude of the effect appears to increase in proportion with the number of squares that moved (points more or less follow the fit line). We evaluated the impact of motion of the individual squares by considering the different random selections of moving squares. The differences (in the direction of background motion) between the finger positions when individual squares did or did not move, differed significantly across the image (F_48,960_ = 2.0; *p*<0.001). The strongest influence of background motion was found near and above the targets (green squares in Fig. 9B). The number of squares that moved, and whether the static or the moving target was small, made no significant difference.

The squares in Fig. 9B are green if they contribute more than average to the influence of background motion, and red if they contribute less than average. Thus, the colours in Fig. 9B tell us which squares were *most* influential, but they do not directly tell us to what extent the influences differ. To examine the differences between the squares’ influences in more detail, we conducted an additional analysis in which we concentrated on the influence of the squares that appeared to be the most important.

**Fig 10:**
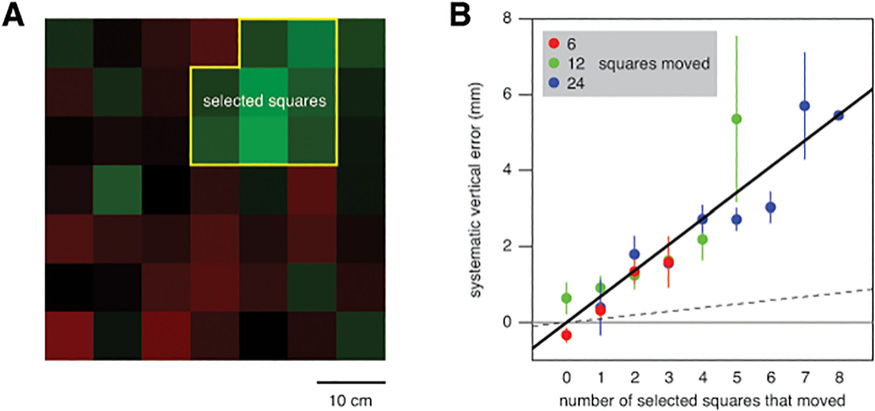
The influence of selected squares. **A**. Reproduction of the squares’ relative influences from Fig. 9B, indicating which squares we selected. **B**. Extent to which the influence of background motion (as shown in Fig. 9A) depended on how many of the selected squares moved. Mean and standard error of subjects’ mean values for each total number of squares that moved (coded by the different colours), with a linear fit to all these points (again, constrained to pass through the origin). The dashed line is the line from Fig. 9A (considering all rather than only the selected squares).

It appears from Fig. 9A that the influence of motion in the background is proportional to the number of squares that moved. It may, however, not be the number of squares (or the surface area) that is important, but only background motion within a limited region near the position that will be tapped (or near where subjects directed their gaze) that is important. Increasing the number of squares that move increases the likelihood that squares within that region move. To distinguish between these possibilities, we selected the squares that appear to have the strongest influence on where people tap (Fig. 10A). Next, we determined how many of these squares had moved on each trial. Then, we determined the influence of background motion for each number of the selected squares that moved, by determining the vertical difference between the average position that was tapped when the background moved upwards and when it moved downwards, and dividing this value by two. This was done separately for each subject and total number of squares that had moved. Finally, we determined the mean and standard error for each combination of the number of total and selected targets that moved (Fig. 10B).

Again, the magnitude of the systematic error appeared to increase in proportion with the number of squares that moved. Obviously, the rate at which the systematic error increased per square that moved was higher for the selected squares (slope of solid line) than for all squares taken together (slope of dashed line), because we selected the squares on the basis of their influence on the error. That the slope is almost 7 times larger when only considering 8 of the 49 squares indicates that the other squares cannot have had much influence. The latter conclusion is more directly evident from the fact that the total number of squares that moved (different colours) hardly influences the relationship between the systematic error and the number of the selected squares that moved. The only clear influence of changing the total number of squares that moved is that there is obviously more data for which many of the selected squares moved when more squares moved. Thus, it would appear that only motion of the squares within the selected region really matters.

## Discussion

Using different sizes for the static and moving targets successfully modulated our subjects’ eye movements in the first experiment (Fig. 4A). When the static target was small, pursuit of the target was negligible. Subjects did occasionally first look to the left, where the large, moving target would appear, but by the time the background moved (250 ms before the targets were to be hit) their eyes were directed at the small, static target. When the moving target was small, subjects did generally pursue it with their eyes, but pursuit was not perfect. In particular, subjects sometimes fixated the large, static target. They initially did so in almost half the trials, but in most cases they then switched their gaze to the small, moving target that they then pursued. We did not exclude trials with poor pursuit, or trials in which subjects made a saccade while the background was moving, because the overall difference in eye movements is evident without doing so, and selecting movements introduces the possibility that both the eye movements and a measure of performance (e.g. tapping error) were influenced by some other factor that also influenced performance when the eyes were not moving. In the latter case trials that were influenced by the other factor would not be excluded. We assume that using different target sizes influenced our subjects’ eye movements similarly in Experiments 2 and 3.

The size of the moving target, and thereby whether or not the eyes were moving, had very little influence on the response to background motion. The background’s influence was just as evident when the moving target was small (so that the eyes were pursuing its motion to the right) as when the static target was small (so that the eyes were not pursuing the moving target). The background’s influence was not particularly strong when the background moved to the left (increasing the retinal slip without changing its direction; Fig. 7A). It was similar for horizontal background motion (along the direction of pursuit; Fig. 7A; Experiment 2) as for vertical background motion (orthogonal to the direction of pursuit; Fig. 4B; Experiment 1). The influence of background motion was strongest when parts of the background moved that were near where the subjects tapped (Figs. 9 and 10; Experiment 3). Moreover, comparing responses when a large numbers of the selected squares moved vertically in Experiment 3 (Fig. 10B) with the responses when all squares moved vertically together in Experiments 1 (Fig. 4B) suggests that, if anything, having static structures near the moving background enhances the response rather than decreasing it. This is more consistent with the response to background motion being a direct response to local motion, than to it arising from a global analysis of the optic flow for estimating and adjusting for self-motion.

Based on previous experimental results, we expected to see the response to the background increase until about 250ms after the background started moving [4,34]. In this study, the average response reached its peak up to 50ms earlier than we had anticipated (Figs. 3 and 6). To make sure that we would not have reached fundamentally different conclusions if we had analysed peak responses rather than final errors, we also analysed the maximal differences between the average paths for backgrounds moving in opposite directions. For each subject, we determined this maximal difference separately for each size and speed of the moving target. For Experiment 1, the overall average peak vertical difference was 10.0 mm. A repeated measures analysis of variance with *target speed* and *which target was small* as factors revealed that the influence of moving the background was larger when the moving target was slow (F_1,9_ = 6.9; *p* = 0.003), irrespective of which target was small. For Experiment 2, the overall average peak horizontal difference was 8.3 mm. Again, the influence of moving the background was larger when the moving target was slow (F_1,9_ = 12.0; *p* = 0.007). In this experiment the influence of moving the background was also larger when the moving target was large (F_1,9_ = 8.3; *p* = 0.02). The interaction was not significant. That analysing the peak differences in this manner yielded a similar pattern of results as the pattern that emerged from our original analysis that was based on the differences at the time of the hit makes us more confident about our original interpretation of the data.

We do not know why the moving background’s influence was consistently larger when the target was moving more slowly (irrespective of whether it was pursued with the eyes). This need not be a direct effect of *target* speed, because when the target was moving faster the time until the targets were aligned was shorter. It might therefore be an effect of the *hand* having moved faster in order to move the required 20 cm in about 30% less time for faster target motion. However, it is not evident why the effect of the background should be smaller when the target or hand is moving faster, so the origin of the effect still needs to be examined.

Moving the whole background influenced the arm similarly in Experiments 1 and 2 as it had when moving towards a static target with no second, moving target indicating when the static target was to be hit [4,20]. The moving background’s influence was also similar to the influence that was found when moving towards the position at which a target had been flashed [26], and when moving towards the position at which a static target had been just before the movement started [22,23,33,34]. Before discussing the small differences between how the moving background influenced the arm in our interception task and in movements towards static, flashed or removed targets, we will first discuss two larger apparent discrepancies between our results and those of previous studies in which the background moved while the eyes were pursuing a moving target that was to be hit.

Moving the background in the direction of—or opposite the direction of—target motion did not influence the mean interception errors in several earlier studies [21,35]. That such background motion did influence tapping errors in the present study (Fig. 7A) could be because we now moved the background at the moment at which it was expected to influence tapping errors most, whereas in the previous studies the background moved longer and started moving earlier. It is evident from Figs. 3 and 6 that the magnitude of the influence of moving the background decreases after the initial response, which suggests that the effect would have been smaller if the background had started moving earlier. However, the difference in the effect of background motion can also be explained by the different measures that were used to evaluate performance. In the earlier studies, subjects were free to intercept the target wherever they liked, so only errors with respect to the moving target were analysed. If we perform additional repeated measures analyses of variance on the mean interception errors of the present study, we find significant effects of target speed, but no significant effects of background motion (or of the size of the moving target, or any interactions). There is therefore no discrepancy between the findings. Apparently, subjects adjust the timing of the hit to where the target will be hit if background motion influences the latter, which might explain why the timing of the tap was influenced by moving the background in the same or opposite direction than the target (Fig. 7B), but not by moving the background in the orthogonal direction (Fig. 4C).

Moving the background in a direction *orthogonal* to the direction of target motion influenced hitting movements in previous studies in which subjects were presumably following the target with their eyes [27,28]. In those studies, the initial direction in which the hand moved was consistent with the background’s influence on the perceived direction of the target’s motion, meaning that the hand deviated in the opposite direction than that in which the background was moving. We found a response in the direction that the background was moving. This difference emphasizes that what we are studying in the present study are fast automatic responses to motion in the surrounding [29], rather than information that is used to predict the target’s future displacement [42–44]. Thus, this apparent discrepancy is probably explained by the different times at which the background was moved. Another difference that may be important is that in the present experiment the target always moved in the same direction, whereas in the earlier experiments the direction in which it moved varied from trial to trial [27,28].

Returning to the studies in which the eyes were not pursuing a moving target, we see an apparent discrepancy between the results of our Experiment 3 and those of Gomi, Abekawa and Shimojo [22]. While we found that only motion nearby the targets was important, they showed that adding motion in the periphery made a difference. They also showed that removing moving structures from near where the target had been makes little difference. Apart from the fact that the target remained visible in our study, whereas it disappeared before the background started moving in their study, a possibly important difference is that in our study the parts of the background that were not moving were clearly visible static structures, whereas in their study motion was removed from parts of the surrounding by removing the texture altogether. Moreover, they mainly found that the effects of background motion were larger for larger backgrounds when the backgrounds were low spatial frequency gratings, whereas we used a background with an undefined (because subjects could stand as they pleased) broad spatial frequency content (a checkerboard pattern). Which of these differences between the studies are responsible for the different spatial sensitivities to background motion remains to be examined.

Abekawa and Gomi [45] also found that moving the background was particularly influential when it occurred in certain regions. They demonstrated that the influence was largest when the visual motion was where gaze was directed, and that the effect was particularly strong when that was also the position at which the target had been. This is consistent with our finding that motion near the targets was critical, given that our subjects’ gaze was always directed at one of the targets.

In a previous study in which moving targets had to be hit at a certain position [3], we found that precision in terms of hitting at the right position (standard deviation of the horizontal position) was highest when the eyes were fixating that position, whereas the precision in terms of being close to the moving target (standard deviation of interception) and the precision in terms of the target being close to where it should have been hit (standard deviation of the timing) were highest when the eyes were following the moving target. In Experiment 1 we also found that the standard deviation of the horizontal position was smaller when the eyes did not pursue the moving target (Fig. 5A), whereas the standard deviation in interception was smaller when the eyes did pursue the moving target (Fig. 5B). The standard deviation in timing was smaller when the eyes pursued the moving target (Fig. 5C), but this effect was not statistically significant.

In Experiment 2 there are trends in the same direction as in Experiment 1, but none of the effects are significant. There are some significant interactions between which target was small (i.e. whether the eyes were moving) and background motion. A logical reason for the advantages and disadvantages of pursuing the moving target with one’s eyes not being as clear in Experiment 2 is that the background moved in the same direction as the target. There was no background motion in the previous study [3]. The background moved in the orthogonal direction to that of the moving target in Experiment 1, so it influenced the vertical errors but not the errors in the direction of target motion. In Experiment 2, the background moved along the moving target’s path, and influenced performance in that direction (Figs. 7 and 8). Performance probably also depended on the condition [41] and error [40] on the previous trial. It seems logical to conclude that all this additional variability that is unrelated to the eye movements overshadowed the advantages and disadvantages of pursuing the moving target in Experiment 2, especially considering that the expected effects were most obvious when the background did not move (Fig. 8). However, the overall variability tends to be smaller, rather than larger, in Experiment 2, so this issue still needs to be resolved.

In Experiment 3 we found that certain parts of the background are particular effective in influencing the arm movement. From Figs. 9 and 10 it would appear that the only squares that mattered were those either directly adjacent to the targets or above the position that was tapped. That moving squares that are above (but not adjacent to) the tapped position influenced the tapping errors, whereas moving squares that are below (but not adjacent to) the targets did not, is probably the result of the latter being close to the hand. Responses to motion in that region may have been suppressed to prevent the hand from responding automatically to its own motion, or to that of related structures such as its shadow. Sometimes, the hand may even have occluded parts of the squares below the tapped position at the time that the background was moving (Fig. 9).

So, should we reject the idea that the fast responses to background motion arise from a mechanism for dealing with self-motion? Not necessarily, because the only conclusion that we can be certain of is that if motion of the background’s retinal image plays a role in updating goal positions in response to self-motion, it does so by relying on local changes near the target rather than by analysing the whole optic flow to separate self from object motion [37,38] or by combining retinal background motion with extra-retinal information about the eye movements [31]. We often interact with objects that are supported by horizontal surfaces or are attached to vertical surfaces, such as cups on tables and light switches on walls, in which case the direct surrounding is close to the object of interest and therefore influenced in a similar manner by self-motion. Responding quickly and automatically to any motion in the vicinity of the target of our actions could therefore often help us cope with our own as well as with obstacles’ motion. In both cases we will not always respond appropriately, but the errors are presumably outweighed by the advantages of responding quickly.

## Supporting Information

S1 File Data used for Fig. 4. Measures are in cm/s, cm and ms.
(TXT)

S2 File Data used for Fig. 5. Measures are in cm and ms.
(TXT)

S3 File Data used for Fig. 7. Measures are in cm and ms.
(TXT)

S4 File Data used for Fig. 8. Measures are in cm and ms.
(TXT)

S5 File Data used for Figs. 9 and 10. Measure is in cm. The values for coordinates xy (each: 1–7) indicate which square moved (1) and which did not (0).
(TXT)
